# Casein Kinase 1δ Activity: A Key Element in the Zebrafish Circadian Timing System

**DOI:** 10.1371/journal.pone.0054189

**Published:** 2013-01-21

**Authors:** Sima Smadja Storz, Adi Tovin, Philipp Mracek, Shahar Alon, Nicholas S. Foulkes, Yoav Gothilf

**Affiliations:** 1 Department of Neurobiology, George S. Wise Faculty of Life Sciences, Tel Aviv University, Tel Aviv, Israel; 2 Sagol School of Neuroscience, Tel Aviv University, Tel Aviv, Israel; 3 Institute of Toxicology and Genetics, Karlsruhe Institute of Technology, Eggenstein-Leopoldshafen, Germany; Tulane University Medical School, United States of America

## Abstract

Zebrafish have become a popular model for studies of the circadian timing mechanism. Taking advantage of its rapid development of a functional circadian clock and the availability of light-entrainable clock-containing cell lines, much knowledge has been gained about the circadian clock system in this species. However, the post-translational modifications of clock proteins, and in particular the phosphorylation of PER proteins by Casein kinase I delta and epsilon (CK1δ and CK1ε), have so far not been examined in the zebrafish. Using pharmacological inhibitors for CK1δ and CK1ε, a pan-CK1δ/ε inhibitor PF-670462, and a CK1ε -selective inhibitor PF-4800567, we show that CK1δ activity is crucial for the functioning of the circadian timing mechanism of zebrafish, while CK1ε plays a minor role. The CK1δ/ε inhibitor disrupted circadian rhythms of promoter activity in the circadian clock-containing zebrafish cell line, PAC-2, while the CK1ε inhibitor had no effect. Zebrafish larvae that were exposed to the CK1δ/ε inhibitor showed no rhythms of locomotor activity while the CK1ε inhibitor had only a minor effect on locomotor activity. Moreover, the addition of the CK1δ/ε inhibitor disrupted rhythms of *aanat2* mRNA expression in the pineal gland. The pineal gland is considered to act as a central clock organ in fish, delivering a rhythmic hormonal signal, melatonin, which is regulated by AANAT2 enzymatic activity. Therefore, CK1δ plays a key role in the circadian timing system of the zebrafish. Furthermore, the effect of CK1δ inhibition on rhythmic locomotor activity may reflect its effect on the function of the central clock in the pineal gland as well as its regulation of peripheral clocks.

## Introduction

Much of what we know today about the molecular mechanisms underlying circadian rhythms in animals can be attributed to detailed studies in the fruit fly and mouse that have employed powerful genetic tools. These studies revealed a core transcription-translation feedback loop that cycles with a circa 24-hour period, and is stabilized by additional auxiliary transcriptional feedback loops. In addition, post-translational modifications of clock components, their stability and sub-cellular localization, contribute to fine tuning the timing of the core loop. These mechanisms operate in almost every cell of multi-cellular organisms and are referred to as ‘peripheral oscillators’. These are synchronized by ‘master oscillators’ such as the clock located in the suprachiasmatic nucleus (SCN) in mammals which represents a specialized structure and which communicates with peripheral clocks by a variety of systemic signals.

Zebrafish have become a popular genetic model and have attracted significant attention from chronobiologists. Detailed studies of the circadian timing mechanism of this species have confirmed existing knowledge and have provided new information regarding the functional development of the circadian clock and its entrainment by light, as well as providing new tools for chronobiological research. One unique feature of zebrafish as a model for circadian biology is the remarkably rapid development of a functional timing mechanism. The pineal gland, considered to function as the master clock in fish, develops by 22 hours post fertilization (hpf), and a circadian clock-controlled rhythm of melatonin production and gene expression begin as early as 2 days post fertilization (dpf) [1]–[4]. These are followed by the appearance of locomotor activity rhythms [5]–[7] and cell cycle rhythms [8] starting during the fifth day of development. It should be noted that the establishment of these rhythms require exposure to light-dark cycles. In transgenic zebrafish, expressing luciferase under the control of the clock gene *period3* promoter, rhythmic luciferase activity in the whole-body was evident on days 5 and 6 post fertilization and depended on light exposure [9]. Importantly, responsiveness to light precedes the formation of the pineal gland or retina [10], [11]. In fact, most zebrafish tissues and even cell lines have clocks that are entrainable by direct exposure to light [12]–[15]. Consequently, cell-based assays have been developed which can be used as an *ex vivo* model system to study the circadian clock and its entrainment by light [16]–[18].

Although much is known about the functional development of the clock system and its entrainment, as well as the genes that compose the core circadian clock in zebrafish, the precise details of the function of this core oscillator remain incompletely understood in this model. For example, the post-translational modifications of clock proteins, and in particular the phosphorylation of PER proteins by Casein kinase I delta and epsilon (CK1δ/ε) have so far not been examined.

CK1 is a family of ser/thr protein kinases which are found in the cytosol, associated with membranes, as well as in the nucleus. In hamsters, a mutation in CK1ε, known as the τ mutation, leads to a short period of 20 hr in homozygous mutants [19], [20]. The *Drosophila* ortholog of CK1ε is called *doubletime* (DBT). Mutations of *doubletime* results in either long (dbt^l^) or short (dbt^s^) circadian rhythms [21], [22]. In humans, a T44A missense mutation in CK1δ is associated with familial advanced sleep phase syndrome (FASPS) [23]. This is characterized by the “morning lark” phenotype, where affected individuals exhibit early sleep times, early-morning awakening, and a shorter period [24]. The effect of these mutations indicates the importance of both CK1δ and CK1ε in period-determining mechanisms within the mammalian circadian clock. Several proteins are phosphorylated by CK1δ/ε, including the mammalian PER1–3 proteins [25], [26]. PER phosphorylation by CK1δ/ε in the cytoplasm induces their translocation to the nucleus. As the PER proteins accumulate in the nucleus, CK1δ/ε phosphorylates them further, leading to their ubiquitin-dependent degradation. Ultimately, the transcriptional repression is fully released, and the genes induced by CLOCK/BMAL1, including *per* and *cry*, are expressed at high levels again. In this way, the circadian clock begins a new cycle [27]. Thus, CK1δ/ε activities play an important role in determining period length.

Here we demonstrate, using a pan-CK1δ/ε inhibitor and a CK1ε-selective inhibitor, that CK1δ activity is crucial for the functioning of the circadian timing mechanism in zebrafish at multiple levels – in peripheral circadian clock-containing cells, in the central clock organ, the pineal gland, and at the organismic level in living animals. In contrast, CK1ε appears to play a relatively minor role in the circadian clock of these systems. In addition, this work demonstrates the utility of commercial CK1 inhibitors for chronobiological research in aquatic model species and emphasizes that zebrafish, larvae and cell lines, represent excellent model systems for high throughput screening for compounds that affect the circadian clock.

## Materials and Methods

### CK1 Inhibitors

Two CK1 inhibitors were used, PF-670462, a pan inhibitor of mammalian CK1δ and CK1ε, and PF-4800567, a selective inhibitor of CK1ε which displays a 22-fold greater potency towards CK1ε than CK1δ (Pfizer Global Research and Development, TOCRIS bioscience) [27]. Both compounds show a high selectivity for CK1 over a panel of other kinases [27]. A 100 mM stock solution was prepared in DMSO and stored at −20°C. Before usage, the stocks were diluted to 100 µM in distilled water and the inhibitors were added to the water that contained the embryos or to cell-culture medium at the indicated concentrations; controls were treated with identical concentrations of DMSO.

### Cell culture, constructs and real-time bioluminescence assays

The PAC-2 cell line [28] was cultured and entrained to 12 h light/12 h dark cycle (LD) cycles as previously described [15], [18]. Stable *per1b-Luc* PAC-2 cell lines were established as described elsewhere [15], [18]. *per1b-Luc* contains the 3.3 kb *per1b* promoter region cloned into the promoterless luciferase reporter vector pGL3Basic (Promega) [15]. Transient transfection of PAC-2 cells with the E-box-Luc construct was done using the FuGene HD reagent according to the manufacturer's protocol (Roche Diagnostics). The *E-box-Luc* construct contains four copies of the *per1b* E-box 5′–CACGTG–3′ cloned into the minimal promoter luciferase reporter vector pLucMCS (Stratagene) [15]. Stably and transiently transfected cells were exposed to various concentrations of the CK1 inhibitors and lighting regimes as indicated and real-time bioluminescence assays were performed and analyzed as described previously [15], [18] using an EnVision multilabel counter (Perkin Elmer).

### Fish and embryos

Adult zebrafish were raised in a recirculating water system under LD cycles at 28°C and fed twice daily [29]. To produce embryos, male and female zebrafish were paired in the evening, and spawning occurred within 1 h of lights-on the following morning. Embryos were placed in 10 cm petri dishes with egg-water containing methylene blue (0.3 p.p.m) and raised under LD cycles at 28°C. To prevent pigmentation (for in situ hybridization analysis, see below), phenylthiourea (PTU) was added to the water during the first two days of development. For locomotor activity analysis, embryos were transferred into a 48 well plate (one larva per well) during the fourth day of development and placed into the DanioVision observation chamber (Noldus Information Technology, the Netherlands). All procedures were approved by the Tel-Aviv University Animal Care Committee and conducted in accordance with the Council for Experiments on Animal Subjects, Ministry of Health.

### Locomotor activity

Zebrafish larval activity was monitored using the DanioVision observation chamber. Larvae were kept under 12 h light/12 h dim light conditions for two days as described [30], inhibitors were then added during the second light phase at the indicated concentrations and larvae were then exposed to constant dim light conditions. Live video tracking was performed using the Ethovision 8.0 software (Noldus information technology). Locomotor activity was measured at days 6–8 post fertilization as the total distance moved by one larva during 10 min time windows. The data is presented as a moving average (10 sliding points) for each group (n = 24).

Fourier analysis was used to test differences in rhythmic locomotor activity using a recently described procedure [30]. The time-dependent signal was converted into a frequency-dependent signal using the Fast Fourier Transform (FFT). The extent to which the original signal of each larva is circadian was quantified by the ratio (‘g-factor’) of the power of the frequency that corresponds to 24 hr period to the sum of powers of all frequencies. The higher the g-factor, the higher is the confidence that the larvae exhibit circadian locomotor activity. Differences in the g-factor distributions between the control and CK1 inhibitor-treated groups were determined by the Kolmogorov-Smirnov test.

### Whole mount in situ hybridization

Samples were collected at different zeitgeber times (ZT, ZT0 corresponding to lights on, ZT12 to lights off) throughout the 24 h cycle, fixed for 24 h in 4% paraformaldehyde and stored in 100% methanol at −20°C. Transcripts of *aanat2, otx5, csnk1δa, csnk1δb* and *csnk1ε* mRNA were detected by whole mount *in situ* hybridization (ISH) using digoxygenin-labeled antisense riboprobes (DIG RNA labeling kit, Roche Diagnostics Ltd, Basel, Switzerland). The *aanat2* and *otx5* probes were produced as previously described [2]–[4], [31]. Whole mount ISH analyses were carried out according to established protocols [31]. The ISH signal, expressed as optical density, was quantified by using ImageJ software (National Institutes of Health, Bethesda, MD, USA). Data were square-root transformed to achieve normality for statistical analysis. Time points were excluded from the statistical analysis when most of the signals were undetectable. Differences in signal intensities between treatments and sampling times were determined by two-way ANOVA. Specific comparisons within each treatment were performed using one-way ANOVA followed by Tukey's post-hoc test. Student's T-test was applied for comparison of single time point data. Results are expressed as mean optical density ± standard error.

## Results

### CK1δ activity is important for zebrafish peripheral clock function

**Figure 1:**
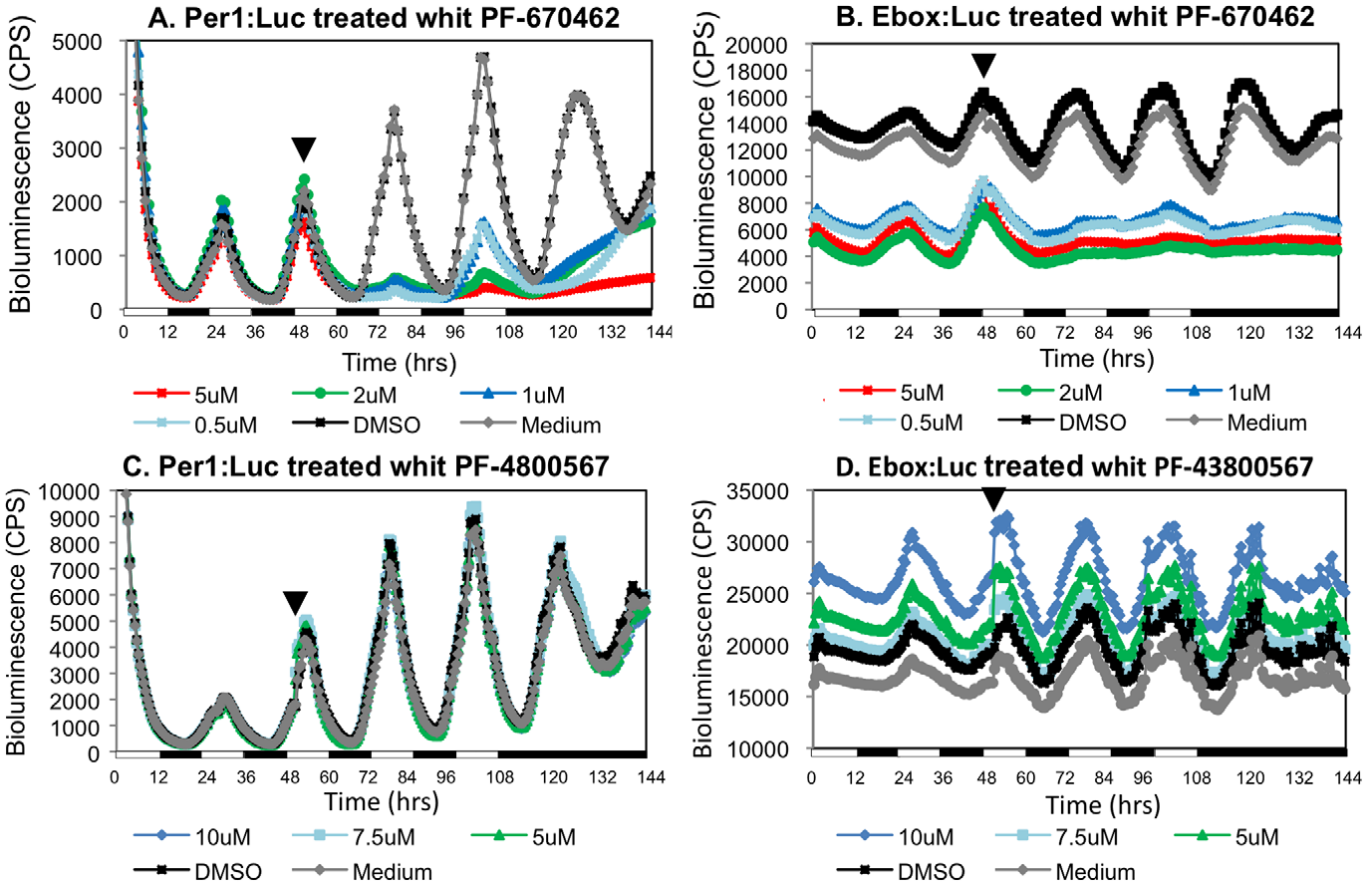
CK1δ inhibition disrupts the PAC-2 cell circadian clock. Dose-response of PF-670462, a pan-CK1δ/ε inhibitor and PF-4800567, a selective inhibitor of CK1ε, in the zebrafish PAC-2 cell line. Bioluminescence assay of cells transfected with per1b:Luc (A and C) or Ebox:Luc (B and D), and treated with PF-670462 (A and B) or with PF-4800567 (C and D) at the indicated time (arrow). Cells were maintained under LD cycles, and then the inhibitor was added to the cell culture 1.5 h before lights on (black arrows) at different concentrations (PF-670462: 0.5 μM- light blue line, 1 μM- blue line, 2 μM-green line, 5 μM- red line. PF-4800567: 5 μM- green line, 7.5 μM- light blue line and 10 μM- blue line). Controls were treated with DMSO (black line) or with culture medium (gray line). After three LD cycles cell were transferred to DD. Bioluminescence is plotted on the y-axis and time (hours) on the x-axis. White/black bars show the light and dark periods, respectively. Clock-controlled rhythmic promoter was disrupted by PF-67046 but not by PF-4800567, thus CK1δ activity appears to be important for peripheral circadian clock function.

To address the contribution of CK1δ/ε to the functioning of the molecular oscillator in peripheral clocks, the effect of treatment with PF-670462 was tested in the zebrafish PAC-2 cell line transfected with the clock-controlled promoter-reporter construct, *per1b-Luc*. These cells contain circadian clocks that are directly entrained by light and exhibit clock-controlled circadian rhythms of luciferase activity upon entrainment by light/dark (LD) cycles, which persist in constant darkness (DD) [17]. Importantly, microarray data [32] indicates that PAC-2 cells express CK1δ but not CK1ε. Therefore, any effect of PF-670462 would reflect CK1δ inhibition. Cells were exposed to 2 LD cycles for entrainment and the inhibitor was then added to the cell culture medium at different concentrations (0.5, 1, 2 and 5 µM) 1.5 h before lights on (Figure 1A). Cells were maintained in LD for additional 3 days and then transferred to DD for 24 h. Luciferase activity was monitored and compared with that of vehicle-treated control cells. By the second LD cycle of treatment the clock-controlled rhythmic activity of the *per1b* promoter was significantly disrupted. At the third LD cycle, treatment with the high dose of PF-670462 (5 µM) completely eliminated the rhythmic *per1b* promoter activity while lower doses of the inhibitor led to a reduction in cycling amplitude (Figure 1A). Under DD conditions no rhythmic expression was detected in the presence of all tested doses of the inhibitor whereas in untreated cells rhythms persisted under DD, as expected. These results indicate that CK1δ activity is essential for circadian clock-driven rhythmic promoter activity in the PAC-2 zebrafish cell line.

The second inhibitor, PF-4800567, which is selective for CK1ε had no effect on the clock-driven rhythmic activity of the *per1b* promoter, even at the highest concentrations (Figure 1C). These results indicate that PF-4800567 does not inhibit zebrafish CK1δ and further strengthen the conclusion that CK1δ activity, and not CK1ε, is essential for circadian clock-driven rhythmic promoter activity in the PAC-2 zebrafish cell line.

It is well accepted that clock-driven rhythmic promoter activity is mediated by the core molecular clock via E-box elements within the *per1b* promoter. To determine whether the CK1 inhibitors disrupt this E-box-mediated activity, we used PAC-2 cells transfected with *E-box-Luc* which has also been shown to exhibit clock-controlled circadian rhythms of luciferase activity upon entrainment by LD cycles, which subsequently persists under DD conditions [15]. Cells were treated as described above and luciferase activity was monitored. As with the *per1b* promoter (Figure 1A), the clock-controlled rhythmic activity of the synthetic E-box promoter was abolished under both LD and DD conditions by all tested concentrations of PF-670462 (Figure 1B), and there was no effect of the CK1ε inhibitor, PF-4800567 (Figure 1D). Again, these results indicate that CK1δ plays a central role in the function of the core molecular clock in the PAC-2 zebrafish cell line.

**Figure 2:**
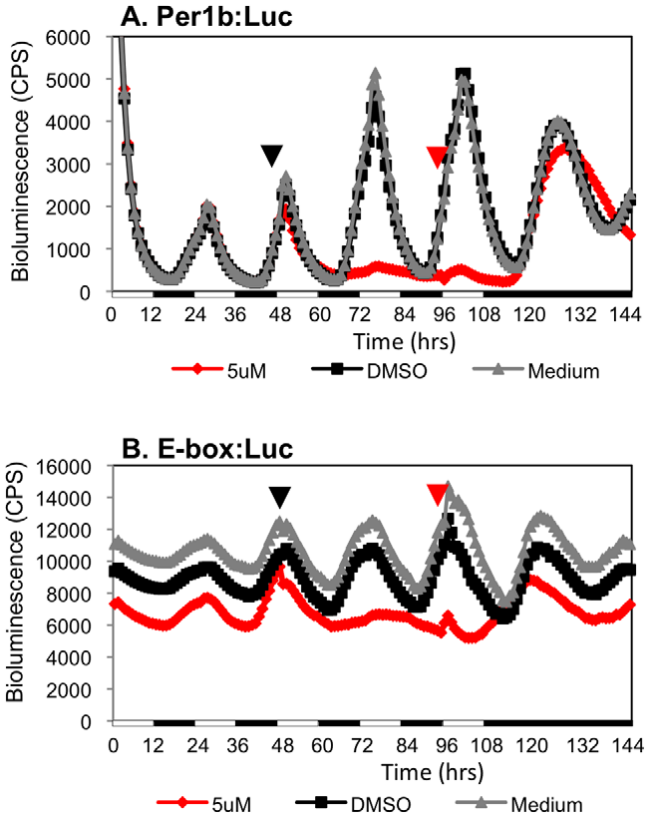
The effect of CK1δ -inhibition on peripheral circadian clocks is reversible. Bioluminescence assay of cells transfected with per1b:Luc (A) and Ebox:Luc (B). Cells were maintained under LD cycles, and then the inhibitor, PF-670462 (5 μM), was added to the cell culture 1.5 h before lights on (black arrows). Cells were maintained in LD conditions for 2 days after which the inhibitor was washed away 3.5 h before lights on (red arrows). After one LD cycle for re-entrainment, the cells were transferred to constant darkness for 24 h. Control cells were treated with DMSO. Bioluminescence is plotted on the y-axis and time (hours) on the x-axis. White/black bars show the light and dark periods, respectively. The clock-controlled rhythmic promoter activity reappeared immediately following removal of the inhibitor. Thus the effects of CK1δ inhibition are reversible.

In order to determine whether the effect of inhibiting CK1δ by PF-670462 is not due to a cytotoxic effect, luciferase activity was analyzed after removal of the inhibitor. PAC-2 cells, transfected with *per1b-Luc* or *E-box-Luc*, were exposed to LD cycles for entrainment, the inhibitor (5 µM) was added before lights on and cells were maintained under LD cycles for 2 additional days. Then, the inhibitor was removed by washing before lights on, the cells were exposed to one LD cycle for re-entrainment and then transferred to DD for 24 h. Importantly, while in the presence of the inhibitor rhythmic activity was abolished, upon removal of the inhibitor both the *per1b* promoter and the synthetic E-box promoter immediately exhibited rhythmic expression under LD and DD conditions (Figure 2). These results indicate that the effect of CK1δ inhibition was reversible, circadian clock-specific, and not due to any general cytotoxic effect.

### Disruption of clock-controlled rhythmic locomotor activity of zebrafish larvae upon CK1 inhibition

**Figure 3:**
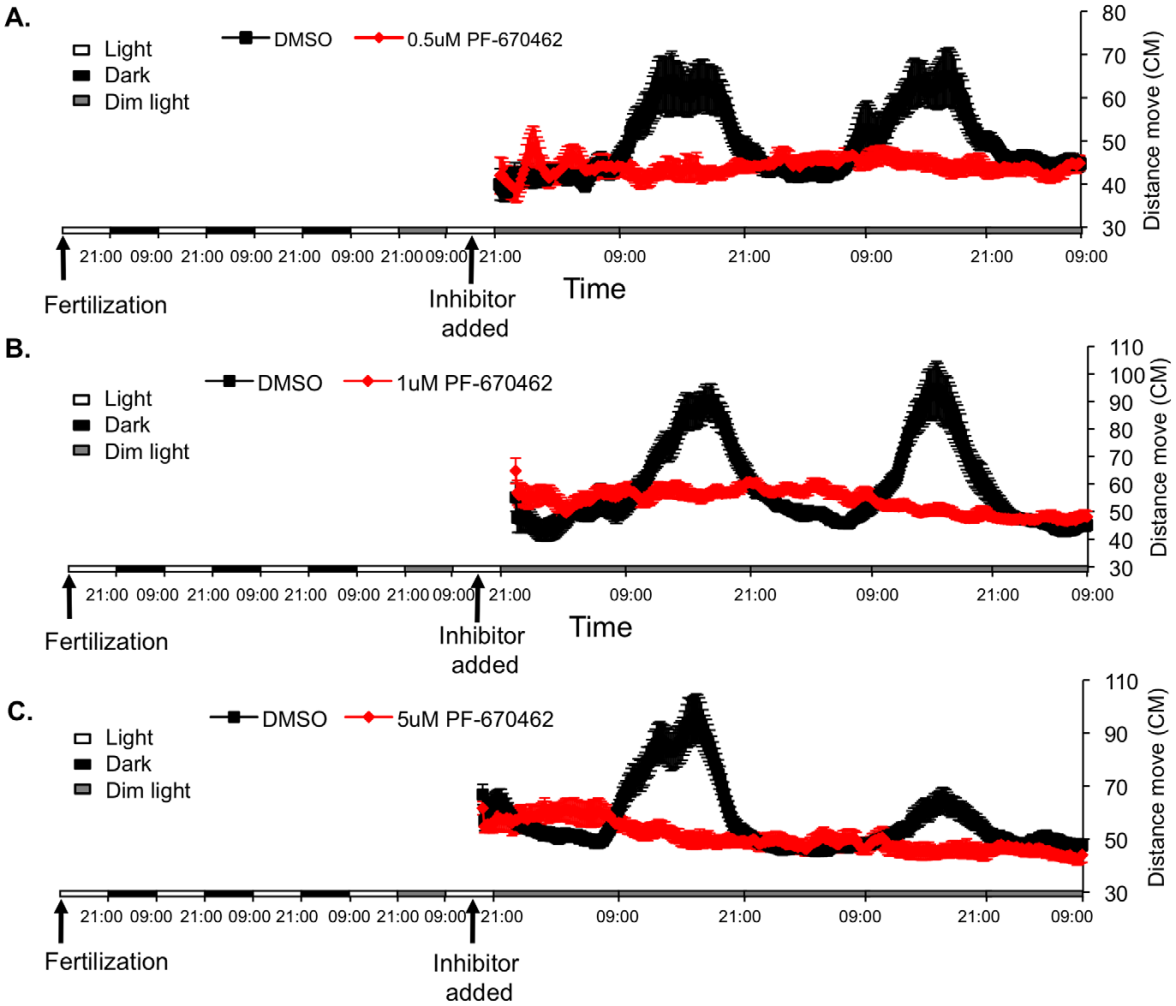
CK1δ inhibition disrupts clock-controlled rhythmic locomotor activity in zebrafish larvae. Locomotor activity of zebrafish larvae was detected by the DanioVision observation chamber. The PF-670462 inhibitor was added to the water at 5 dpf at different concentrations, A-0.5 μM, B-1 μM and C-5 μM (red lines). Control larvae were treated with DMSO at the same concentrations (black lines). The larvae were kept under constant conditions (dim light) and the distance moved (cm, y-axis) was recorded over time (hours, x-axis). The horizontal bars represent the light conditions before and during the experiment. White boxes represent light, black boxes represent dark and grey boxes represent dim light. The rhythm of locomotor activity was completely abolished by the CK1δ inhibitor.

**Figure 4:**
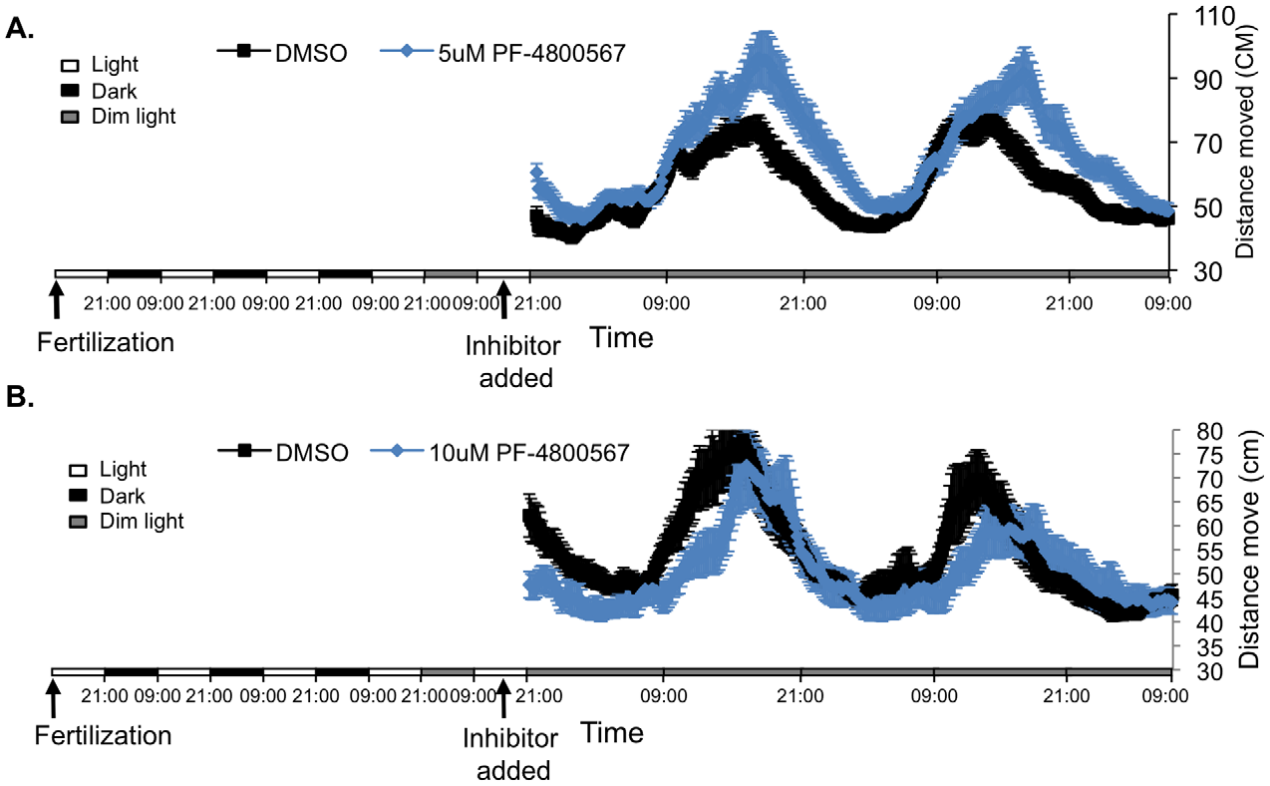
CK1ε inhibition does not affect clock-controlled rhythmic locomotor activity in zebrafish larvae. Locomotor activity of zebrafish larvae was detected by the DanioVision observation chamber. Distance moved (cm) is plotted on the y-axis and time (hours) on the x-axis. The horizontal bars represent the lighting conditions before and during the experiment. White boxes represent light, black boxes represent dark and grey boxes represent dim light. The PF-480567 inhibitor was added to the water at two different concentrations, A-5 μM and B-10 μM (blue lines). Control larvae were treated with DMSO at the same concentrations (black lines). CK1ε inhibition did not affect the locomotor activity rhythm, but did have a slight phase shifting effect.

The impact of pharmacological inhibition of CK1δ and CK1ε isoforms, by using PF-670462 and PF-4800567, was next tested *in vivo* by analysing locomotor activity of zebrafish larvae. After 2 days exposure to light:dim-light cycles, control untreated embryos exhibited circadian rhythms of locomotor activity under constant dim light, as expected. Treatment with 0.5, 1 and 5 μM of the CK1δ/ε inhibitor, PF-670462, abolished these rhythms and the larvae exhibited intermediate levels of activity (Figure 3). The disrupted circadian activity pattern of individual larvae is confirmed using Fourier analysis (p<0.001 by Kolmogorov-Smirnov test; Figure S1A; see also ‘Locomotor activity’ in ‘Materials and methods’); none of the PF-670462-treated larvae exhibit a 24 h-period signal that surpasses the median signal for the control larvae. In contrast, Larvae that were treated with the CK1ε- selective inhibitor, PF-4800567, exhibited rhythmic locomotor activity at all tested doses and there were no significant differences between control and treated larvae (Kolmogorov-Smirnov test; Figure S1B; see also ‘Locomotor activity’ in ‘Materials and methods’). Nevertheless, the PF-4800567-treated groups seem to exhibit a minor phase shift in their locomotor activity rhythm (Figure 4). Although the data, spanning only two days, does not permit statistical confirmation of a phase shift, it may reflect a role for CK1ε in phase re-setting, and possibly entrainment of the clock. Thus, these results indicate that while CK1ε seems to have little effect, CK1δ plays a key role in the circadian clock mechanism of zebrafish.

To confirm that PF-670462 does not simply impair larval motility, locomotor activity levels were tracked under light-to-dark transitions [33]. On day 6 post fertilization, control (DMSO-treated) and PF-670462-treated (5 μM) groups (n = 24) were subjected to 3 dark flashes of 10 sec each during the light phase. Activity was measured as the distance moved by each larva during the dark flash. No statistical difference was observed between the activity of the control and the PF-670462-treated groups (Student's *t*-test, P-value>0.5; Figure S2), indicating that the PF-670462 at the highest concentration used (5 μM), does not impair larval locomotor activity.

### CK1δ activity is important for the functioning of the central clock

**Figure 5:**
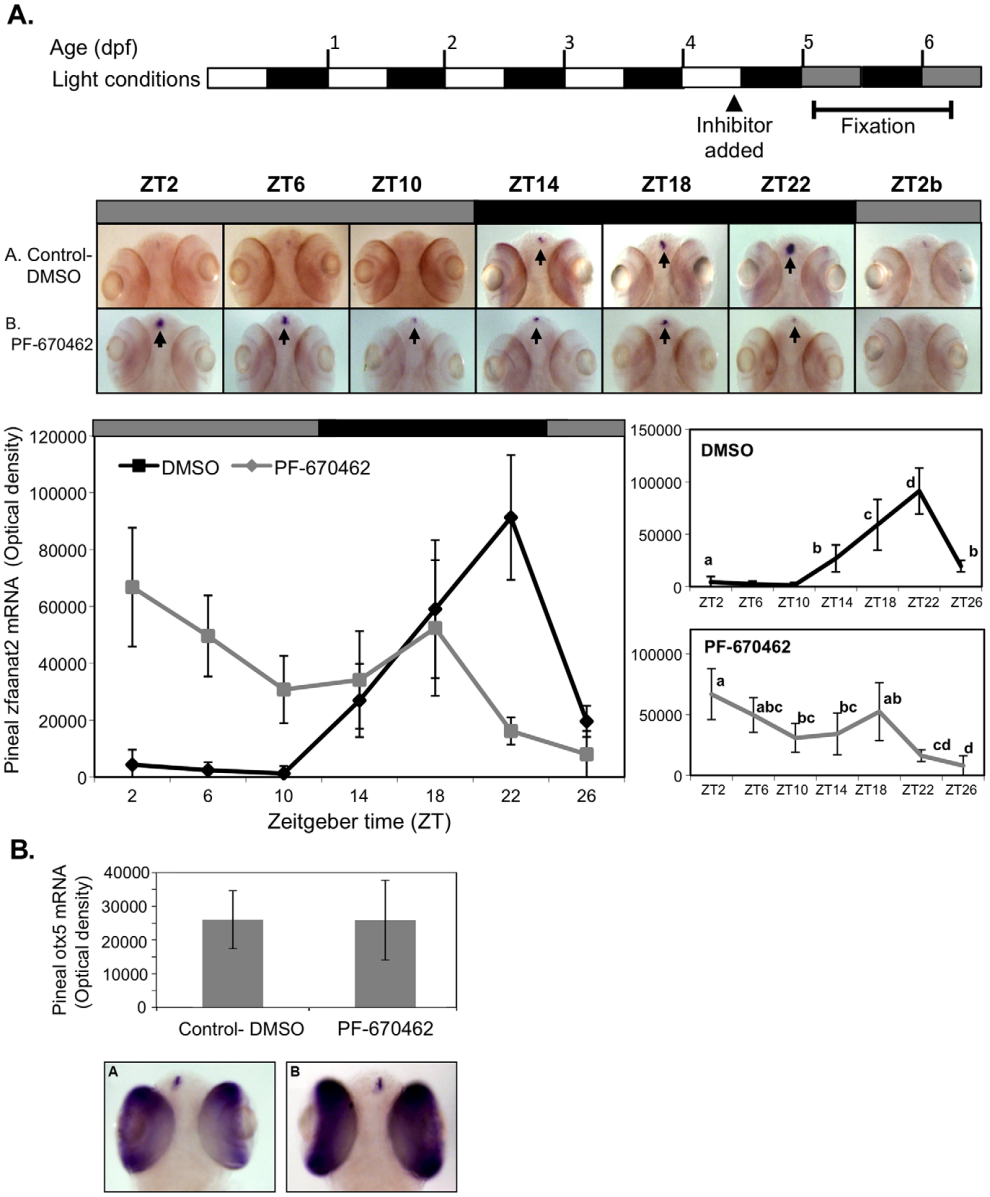
CK1δ inhibition abolishes rhythmic pineal *aanat2* mRNA expression. A. Top panel: Schematic representation of the experimental design. The horizontal bars represent the light conditions before and during sampling; white boxes represent light, grey boxes represent subjective day and black boxes represent dark. Middle panel: Whole-mount ISH signals for *aanat2* mRNA (dorsal views of the heads) of representative specimens treated with DMSO (control, a) or with PF-670462 (5 μM) (a). Zeitgeber times (ZT) are indicated for each sample. ZT0 corresponds to “lights-on,” ZT12 to “lights-off”. ZT2b refers to the second day of the experiment. ISH signals in the pineal gland are indicated by arrows. Bottom chart: Quantification of signal intensities in the pineal glands of control (DMSO) larvae (black line) and PF-670462-treated larvae (grey line). Values represent the mean ± SE optical densities of the pineal signals. *Aanat2* mRNA expression is significantly affected by treatment and sampling times (P<0.01 by two way ANOVA). Differences in sampling time within each treatment were determined by one-way ANOVA followed by Tukey's post-hoc test for each treatment. B. Whole-mount ISH signals for *otx5* mRNA at ZT2. Photographs of pineal signals in (a) DMSO (control) and (b) CK1δ-treated larvae are presented in the bottom panel.

Given the striking effect of the pan-CK1δ/ε inhibitor PF-670462 on rhythmic locomotor activity, its effect on the central circadian clock organ, the pineal gland, was of interest. Notably, data mining of deep sequencing data [30] indicates that while CK1δ mRNA is expressed at high levels, CK1ε mRNA is expressed at very low levels in the pineal gland (the lower quartile of all genes). Thus we investigated the role of CK1δ in the pineal gland by testing its effect on rhythmic expression of *arylalkylamine-N-acetyltransferase2* (*aanat2*). Zebrafish *aanat2* is expressed exclusively in the pineal gland and exhibits a robust rhythmic expression pattern as early as 2 days post fertilization (dpf). It has therefore been used as a marker for pineal circadian clock function in the zebrafish [2]–[4], [30]. Embryos were kept under LD cycles for 5 days. On the fifth day the inhibitor (PF-670462, 5 μM) was added to the water before lights off (Figure 5A top panel). Embryos were then exposed to DD and were collected at 4 h intervals during the sixth and seventh day of development, and *aanat2* mRNA levels were measured by ISH. The control group exhibited a robust pineal *aanat2* mRNA rhythm with high levels at night and low levels during the subjective day (Figure 5A), as was previously reported [2]–[4], [31]. In contrast, embryos that were treated with the inhibitor did not display any rhythmic expression but, rather, intermediate levels of pineal *aanat2* mRNA throughout the day. Thus, inhibition of CK1δ had a drastic effect on clock-controlled rhythmic gene expression in the pineal gland, indicating that CK1δ is essential to the function of the central circadian clock.

To control for any possible developmental or morphological effects of the inhibitor, we performed whole mount ISH for *otx5*, a pineal and retinal gene that displays an arrhythmic expression pattern [3], [31]. This control ISH was performed on embryos that were collected at ZT2, a time in which *aanat2* levels differ dramatically between treated and control larvae (p-value<0.05, student T test). Both experimental and control groups exhibit the same level of *otx5* expression (Figure 5B) (p-value>0.05, student T test), indicating that the pineal morphology was not affected by the inhibitor.

**Figure 6:**
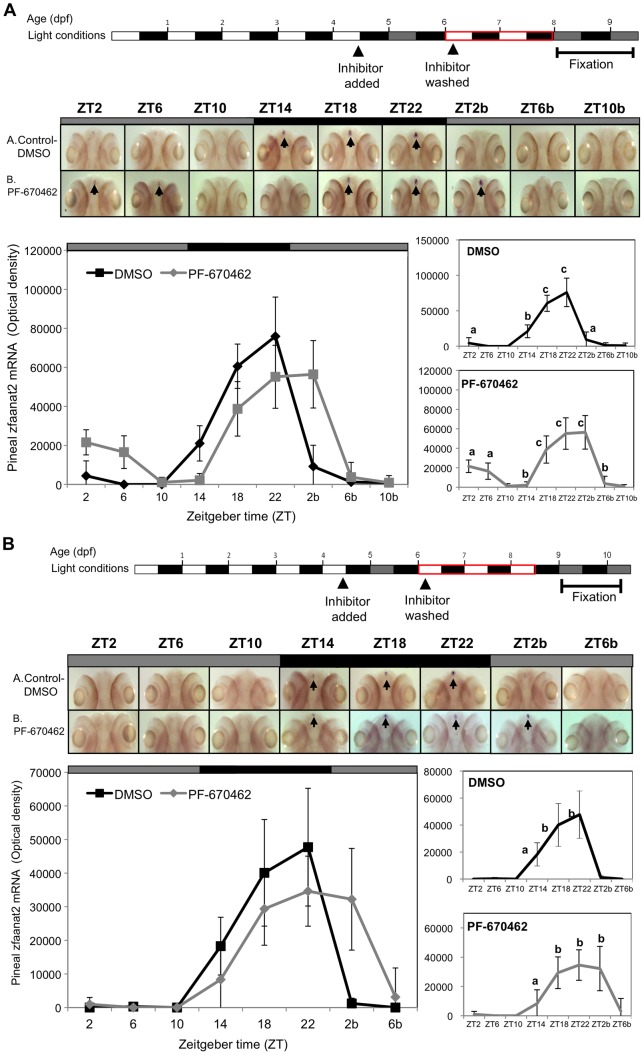
The effect of CK1δ inhibition on rhythmic pineal *aanat2* mRNA expression is reversible. Reversibility was determined after two (A) or three (B) LD cycles for re-entrainment. **Top panels**: Experimental design. The horizontal bars represent the light conditions before and during sampling; white boxes represent light, grey boxes represent subjective day and black boxes represent dark. Middle panels: Whole-mount ISH signals (dorsal views of the heads) of representative specimens treated with DMSO (control, a) or with PF-670462 (b). ZT 2-10b refers to the second 24 h cycle of the sampling. Bottom panels: Signal intensities in the pineal gland. Values represent the mean ± SE optical density of the pineal signals. Statistical analysis was performed using one-way ANOVA followed by Tukey's post-hoc test for each treatment. Following removal of the inhibitor and re-entrainment, for two (A) or three (B) LD cycles, the rhythm of aanat2 mRNA re-appeared but seemed to be shifted.

Given the striking effect of CK1δ inhibition on the pineal gland circadian clock, we asked whether this effect could be rescued as shown above for a peripheral clock. To address this question, we performed the following experiments. Embryos were kept under LD cycles during the first 4 days of development and the inhibitor was added to the water on the fourth day before lights off (Figure 6A top panel). Larvae were then placed in DD and sampled at 4 h intervals during the fifth day of development to confirm arhythmicity. On the sixth day of development the inhibitor was removed by washing. Subsequently, larvae were exposed to two or three LD cycles (figure 6A and B, respectively) for re-entrainment, transferred to DD again, and then sampled every 4 h for ISH analysis of *aanat2* mRNA levels. Following removal of the inhibitor and re-entrainment, rhythmic *zfaanat2* mRNA expression was detected in both control and treated groups (two way ANOVA followed by Tukey's post-hoc test). We note that the rhythms in PF-670462-treated groups seem to be shifted after re-entrainment (figure 6A and B). Together these results indicate that the effect of the inhibitor on the functioning of the central circadian clock is reversible, and is likely to be clock-specific.

## Discussion

The role of CK1δ and CK1ε in the circadian clock has been studied in *Drosophila* and mammals [34]–[37], but to date little is known about the contribution of these two enzymes to circadian clock function in non-mammalian vertebrates. In the present study we reveal an essential role for CK1δ activity in the circadian clock mechanism in zebrafish at multiple levels – in peripheral circadian clock-containing cells; in the central clock organ, the pineal gland; and at the whole organism level. Our results indicate that inhibition of CK1δ has a drastic effect on the function of the core molecular clock in the PAC-2 zebrafish cell line, as indicated by the complete elimination of rhythmic transcription directed by the *per1b* promoter and the synthetic E-box promoter. CK1δ inhibition also abolishes the clock-controlled rhythmic expression of *aanat2* mRNA in the pineal gland, which is considered a central clock organ in the zebrafish, and consistently, completely eliminates the rhythmic locomotor activity rhythms of living zebrafish larvae. Importantly, our results reveal that these effects are reversible and are likely clock-specific. In contrast, inhibition of CK1ε had no effect on the molecular clock in the peripheral clock-containing PAC-2 cells and minor phase shifting effect on the clock-controlled rhythmic locomotor activity of zebrafish larvae. This effect, although not statistically significant, warrants further investigation with a focus on the entrainment and phase shifting of the clock. Nevertheless, our results point to a predominant role of CK1δ in the zebrafish circadian clock system.

The compounds used in the current study were originally designed to inhibit human CK1δ and CK1ε, and *in vitro* data on their capacity to inhibit the zebrafish CK1δ and CK1ε is not available. Nevertheless, alignment of the human and zebrafish CK1δ and CK1ε sequences reveals that the catalytic domains are 99% and 97% identical, respectively, and that the minor differences are in amino acid residues that are not crucial for the enzymatic activity nor are involved in interaction with the inhibitors [38]. Therefore, it is likely that the inhibitors have similar specificities in human and zebrafish. This is also reflected in our data: As indicated in the ‘Results’. CK1δ mRNA is expressed at high levels in PAC-2 cells while CK1ε mRNA is not [32]. The fact that PF-4800567 did not affect rhythmic promoter activity in PAC-2 cells is therefore an indication that this compound does not inhibit CK1δ activity and is likely CK1ε-selective, as was shown for the human enzymes [27].

Mining of zebrafish microarray [32], [39] and deep sequencing data [30], indicates that while CK1δ mRNA is expressed in the brain, as well as in PAC-2 cells and in the pineal gland, CK1ε mRNA is not expressed in PAC-2 cells, is expressed at very low levels in the pineal gland (the lower quartile of all genes), and at moderate levels in the brain. Consequently, it is tempting to speculate that the possible phase shifting effect of the CK1ε inhibitor (PF-4800567) on rhythmic locomotor activity in living larvae is mediated by an extra-pineal brain structure where CK1ε is expressed. Thus, the widespread distribution of CK1δ as compared with CK1ε, its rhythmic expression in the pineal gland (Figure S3) and the major effects of its inhibition on the peripheral and central clocks and rhythmic locomotor activity, indicate that CK1δ plays a key role in circadian timing system of the zebrafish. The effect of CK1δ inhibition on rhythmic locomotor behavior may be the result of its effect on the central clock in the pineal gland as well as its effect on peripheral clocks.

The precise effects of the CK1 inhibitors, PF-4800567 and PF-670462, seem to be species-specific. A work by Walton et al showed that the pan-CK1δ/ε inhibitor (PF-670462) dramatically slows rhythms of PER2:LUC bioluminescence in rat fibroblasts *in vitro* in a dose-dependent manner (0.1–10 µM), while the CK1ε inhibitor (PF-4800567) had no effect at even higher concentrations (0.03–30 µM) [27]. Furthermore, an *in vivo* study of mice locomotor activity showed that administration of the pan-CK1 δ/ε inhibitor delayed the active period by 6.5 h, while inhibition of CK1ε caused only a small phase shift of 0.5 h [27]. A recent study by Meng et al showed that high doses of the pan-CK1δ/ε inhibitor (10 µM) caused persistent period lengthening with eventual loss of rhythmicity within the SCN of wild type mice [36]. In the current study, however, inhibition of CK1ε had only a minor phase shifting effect and the use of a pan-CK1δ/ε inhibitor (PF-670462) did not lengthen the period but rather abolished the rhythm at multiple levels. The reasons for these species-specific effects may be related to differing experimental conditions (i.e. doses and timing) or may reflect true differences that are due, for example, to the presence of two CK1 δ isoforms, CK1 δ a and CK1 δ b, in the zebrafish. This possibility calls for further investigation of the relative functional contribution of each CK1δ isoform. Nonetheless, lengthening of the rhythm of zebrafish *per3* promoter activity was observed in transgenic per3:luciferase larvae [9] when exposed to a different CK1 inhibitor, longdaysin [40]. However, it should be noted that this compound also inhibits CK1α and ERK2, and that its relative effect on the two CK1δ isoforms remains unknown.

An important aspect of the current study is that the CK1 inhibitors were applied *in vivo* non-invasively by simply adding them to the water. This approach may be now used to investigate the role of CK1 in other aquatic species in which the circadian clock or tidal clocks are being investigated. Recent examples include the marine isopod *Eurydice pulchra* [41], corals (*Acropora millepora* [42], [43]; *Favia Fragum* [44]), sea anemone (*Nematostella vectensis* [45]) and tunicates (*Ciona intestinalis* [46]), in which functional gene analysis is still lacking.

In addition to being particularly amenable to genetic manipulation, which made it a vertebrate of choice for many aspects of biomedical research, the zebrafish has been successfully exploited as a model for high throughput screening. The current study demonstrates the effectiveness of using zebrafish for testing pharmacological compounds which target the circadian clock, both *in vitro* and, non-invasively, *in vivo*. The ability to use zebrafish in high throughput screens for circadian clock-related drugs opens the way for future treatment of physical and mental disorders that are caused by disruption of circadian rhythms.

## Supporting Information

Figure S1 **Distribution of g-factor values of control and CK1 inhibitor-treated larvae.** Significant differences in the g-factor distribution were revealed between the DMSO (black dots) and PF-670462 (grey dots) treated groups at all tested concentrations (0.5–5 µM) (A) (Kolmogorov-Smirnov test, P-value<0.002). No differences were detected between the DMSO (black dots) and PF-4800567 (blue dots) treated groups at all tested concentrations (5–10 µM) (B) (Kolmogorov-Smirnov test, P-value = 0.6). The median is represented for each group (red line).
(TIF)

Figure S2 **Locomotor activity levels in response to 3 dark flash stimuli.** On day 6 post fertilization, PF-670462 treated (grey bars) and control (DMSO treated, blue bars) larvae (n = 24) were subjected to 3 dark flashes of 10 sec each during the light phase. Each bar represents the average of the 3 dark flashes. Activity was measured as the average distance moved in 1 sec time bins. Error bars represent SE (n = 24). Black and white horizontal boxes represent light phase and dark flash, respectively.
(TIF)

Figure S3 **Temporal expression patters of mRNAs encoding CK1 enzymes in the zebrafish pineal gland, determined by RNA-seq analysis** [**30**] **.** CK1δa and CK1δb mRNAs expression patterns are shown in black and grey lines, respectively, and CK1ε is shown in blue. CT =  circadian time. Gray and black bars represent subjective day and subjective night, respectively.
(TIF)
