# Supplementary figures and images for: Casein Kinase 1δ Activity: A Key Element in the Zebrafish Circadian Timing System
Source: PLoS One. 2013 Jan 21;8(1):e54189. doi: 10.1371/journal.pone.0054189 (PMC3549995; doi:10.1371/journal.pone.0054189)

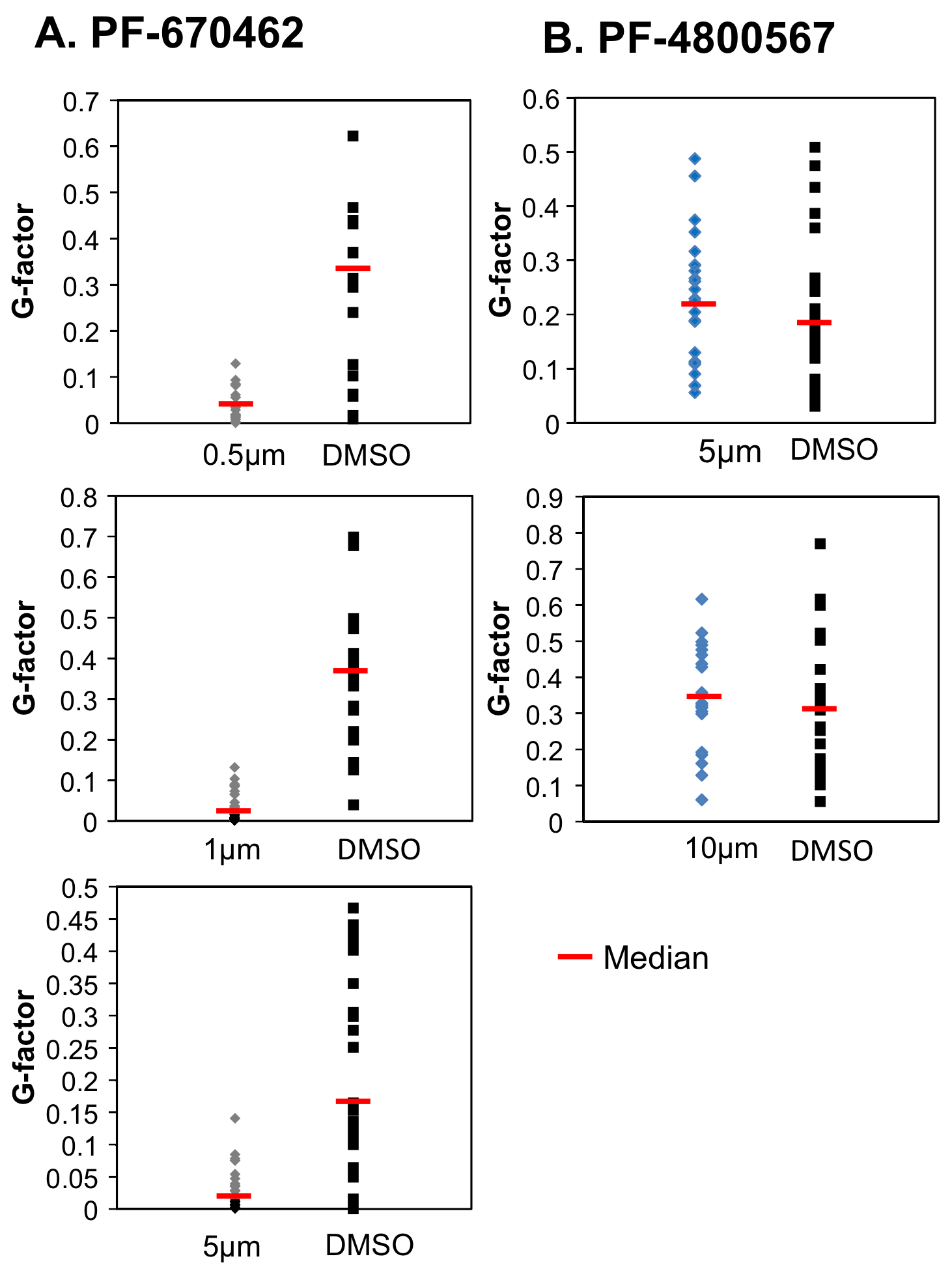

Supplement: Figure S1 — Distribution of g-factor values of control and CK1 inhibitor-treated larvae. Significant differences in the g-factor distribution were revealed between the DMSO (black dots) and PF-670462 (grey dots) treated groups at all tested concentrations (0.5–5 µM) (A) (Kolmogorov-Smirnov test, P-value<0.002). No differences were detected between the DMSO (black dots) and PF-4800567 (blue dots) treated groups at all tested concentrations (5–10 µM) (B) (Kolmogorov-Smirnov test, P-value = 0.6). The median is represented for each group (red line). (TIF) [file pone.0054189.s001.tif]

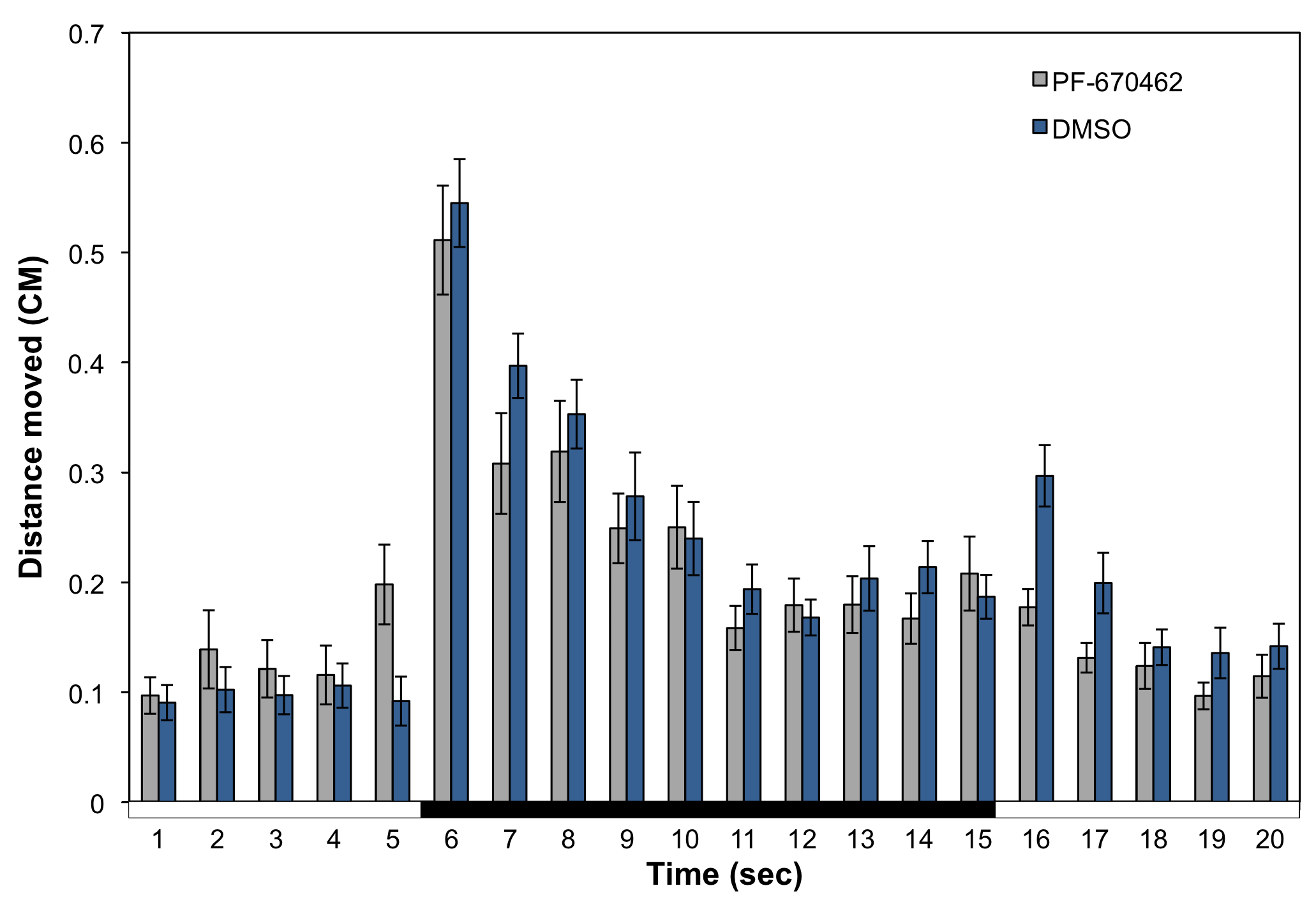

Supplement: Figure S2 — Locomotor activity levels in response to 3 dark flash stimuli. On day 6 post fertilization, PF-670462 treated (grey bars) and control (DMSO treated, blue bars) larvae (n = 24) were subjected to 3 dark flashes of 10 sec each during the light phase. Each bar represents the average of the 3 dark flashes. Activity was measured as the average distance moved in 1 sec time bins. Error bars represent SE (n = 24). Black and white horizontal boxes represent light phase and dark flash, respectively. (TIF) [file pone.0054189.s002.tif]

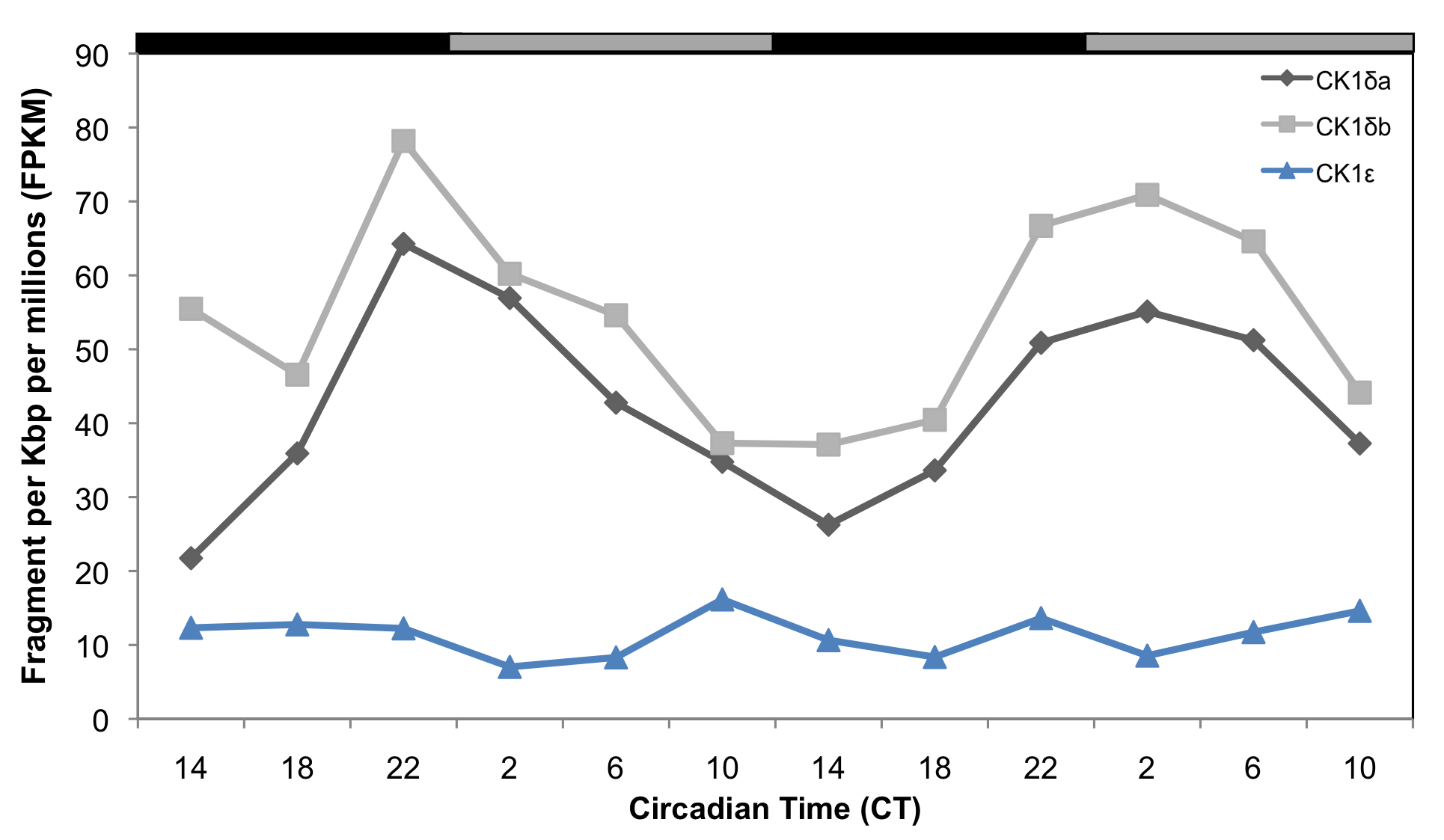

Supplement: Figure S3 — Temporal expression patters of mRNAs encoding CK1 enzymes in the zebrafish pineal gland, determined by RNA-seq analysis [30] . CK1δa and CK1δb mRNAs expression patterns are shown in black and grey lines, respectively, and CK1ε is shown in blue. CT = circadian time. Gray and black bars represent subjective day and subjective night, respectively. (TIF) [file pone.0054189.s003.tif]
